# Outcome of Preterm Neonates > 32 Weeks Gestation in Relation to Three-Tiered Fetal Heart Rate Categorization

**DOI:** 10.3390/medicina61071171

**Published:** 2025-06-28

**Authors:** Jelena Sabljić, Klara Čogelja, Edita Runjić, Blagoja Markoski, Marijana Barbača, Toni Modrić, Boris Bačić

**Affiliations:** 1 Department of Neonatology, Split University Hospital Centre, 21000 Split, Croatia; jsabljic@kbsplit.hr (J.S.); klara_cogelja@yahoo.com (K.Č.); 2 School of Medicine, University of Split, 21000 Split, Croatia; editarunjic@gmail.com (E.R.); marijanabarbaca22@gmail.com (M.B.); borisbacicstdr@gmail.com (B.B.); 3 Department of Pediatrics, Split University Hospital Centre, 21000 Split, Croatia; 4 Department of Obstetrics and Gynecology, Split University Hospital Centre, 21000 Split, Croatia; markoski95@gmail.com; 5 Faculty of Kinesiology, University of Split, 21000 Split, Croatia

**Keywords:** fetal heart rate, cardiotocography, neonates, fetal growth restriction, neonatal respiratory distress syndrome, fresh frozen plasma, oligohydramnios

## Abstract

*Background and Objectives:* Electronic fetal heart rate monitoring is mandatory for preterm labor. Moderate to late preterm neonates have an increased risk of overall morbidity, neonatal intensive care (NICU) admission, and consequently, medication use. The outcome of preterm neonates > 32 weeks of gestation in relation to three-tiered fetal heart rate (FHR) categorization was analyzed. *Materials and Methods:* This was a single-center, retrospective case-control study conducted from January 2021 to December 2023. The study included 25 FGR and 131 control cases born from 33 to 36 6/7 gestational weeks. Outcome was defined as the need for assistance after birth in first 15 min of life, respiratory outcome, and first day dopamine use and fresh frozen plasma transfusion. Maternal characteristics as risk factors for non-normal categories within three-tiered FHR categorization were also analyzed. *Results:* There was no significant difference in neonatal outcome among groups, except significantly lower 1 min APGAR and longer LOS in the FGR group. An increasing category within the three-tiered FHR categorization positively correlated with the need for assistance after birth, respiratory outcome, dopamine use, fresh frozen plasma transfusion, and length of hospital stay. Negative correlations were revealed between the increasing category within the three-tiered FHR categorization and first and fifth minute APGAR scores. Oligohydramnios and male sex were risk factors for non-normal categories within three-tiered FHR categorization. The correlation was tested using the Spearman correlation coefficient. A logistic regression model was employed to identify maternal risk factors for the non-normal category within three-tiered FHR categorization. All differences were statistically significant (*p* < 0.05). *Conclusions:* The increasing category within three-tiered FHR categorization may alert neonatologists to be highly suspicious of RDS, respiratory support, dopamine use, and fresh frozen plasma transfusion in neonates born from 33 to 36 6/7 gestational weeks. Oligohydramnios and male sex increase the probability for non-normal categories in the three-tiered FHR categorization.

## 1. Introduction

Moderate to late preterm neonates have an increased risk of overall morbidity, neonatal intensive care (NICU) admission, and consequently medication use compared to term neonates [1,2,3,4,5,6]. Electronic fetal heart rate (FHR) monitoring is mandatory in preterm labor. Electronic FHR monitoring involves the use of a cardiotocograph (CTG) to record the FHR to determine fetal well-being to detect signs of intrapartum hypoxia [7]. Recently, in a systematic review and meta-analysis, Zullo et al. evaluated the impact of different FHR patterns on neonatal outcomes. They showed that a three-tiered FHR tracing interpretation system provides an approximate but imprecise measurement of neonatal prognosis in term neonates; the majority of neonates with category II or III tracings do not have adverse outcomes (fifth min APGAR score < 7 or umbilical artery ph < 7.00, neonatal seizures, hypoxic ischemic encephalopathy) [8]. A systematic catalog of studies of FHR patterns observed an increase in abnormal FHR patterns in neonates with hypoxic ischemic encephalopathy (HIE), including preterm neonates, although predictive ability was found to be limited [9]. Published studies on CTG in preterm neonates are scarce [10]. Respiratory morbidity as a neonatal outcome was frequently analyzed, and it was associated with abnormal FHR, studied mostly in term neonates [11,12,13,14]. Studies regarding the risk of respiratory distress syndrome (RDS) in fetal growth restriction (FGR) neonates compared to non-FGR neonates continuously report equivocal evidence [15,16,17,18]. Torrance et al. reported that abnormal FHR predicted RDS in a study of 180 FGR neonates born before 34 weeks gestation [19].

Preterm neonates often have hypotension, which may be due to various etiologies; therefore, vasoactive medications are used to provide cardiovascular support [20]. Fresh frozen plasma transfusions are common practice in the neonatal intensive care unit (NICU) [21]. The first days after preterm birth are a critical period of cardiovascular instability, where hypotension is common. Golder has shown that, in preterm neonates who were deemed clinically hypotensive and received inotropic support, the sympathovagal balance was lower, suggesting that autonomic control of heart rate in these neonates was impaired compared to gestational age-matched non-hypotensive infants [22]. The first autonomic cardiovascular function evaluation is performed during cardiotocography, antenatally.

Fetal heart rate patterns remain a critical index of fetal wellbeing throughout gestation and during labor, despite poor positive predictive value for fetal compromise [23]. Intrapartum hypoxia, detected by intrapartum cardiotocography, may lead to alterations in the fetal central nervous system that directly affect the electrical activity of the fetal heart [23,24,25]. Therefore, FHR may be an early predictor of multiple neonatal complications consequent to hypoxia and may influence postnatal care and management.

We aimed to investigate the differences in neonatal outcomes between the FGR and control groups, as well as the correlation between three-tiered FHR categorization and respiratory outcomes, the use of vasoactive medications, and fresh frozen plasma transfusions in moderate to late preterm neonates admitted to level III NICU.

## 2. Materials and Methods

This was a retrospective case-control study of moderate to late preterm neonates with and without FGR from singleton pregnancies born between 1 January 2021 and 12 December 2023, in a single tertiary hospital center in Croatia, University Hospital Centre Split, University Clinic for Gynecology and Obstetrics, Neonatology Department. Maternal and neonatal data were retrieved manually by study personnel from the center’s computerized database and the center’s paper medical records. The retrieved data included maternal and neonatal characteristics, perinatal medical history, and discharge letters, respectively.

### 2.1. Study Population

**Figure 1:**
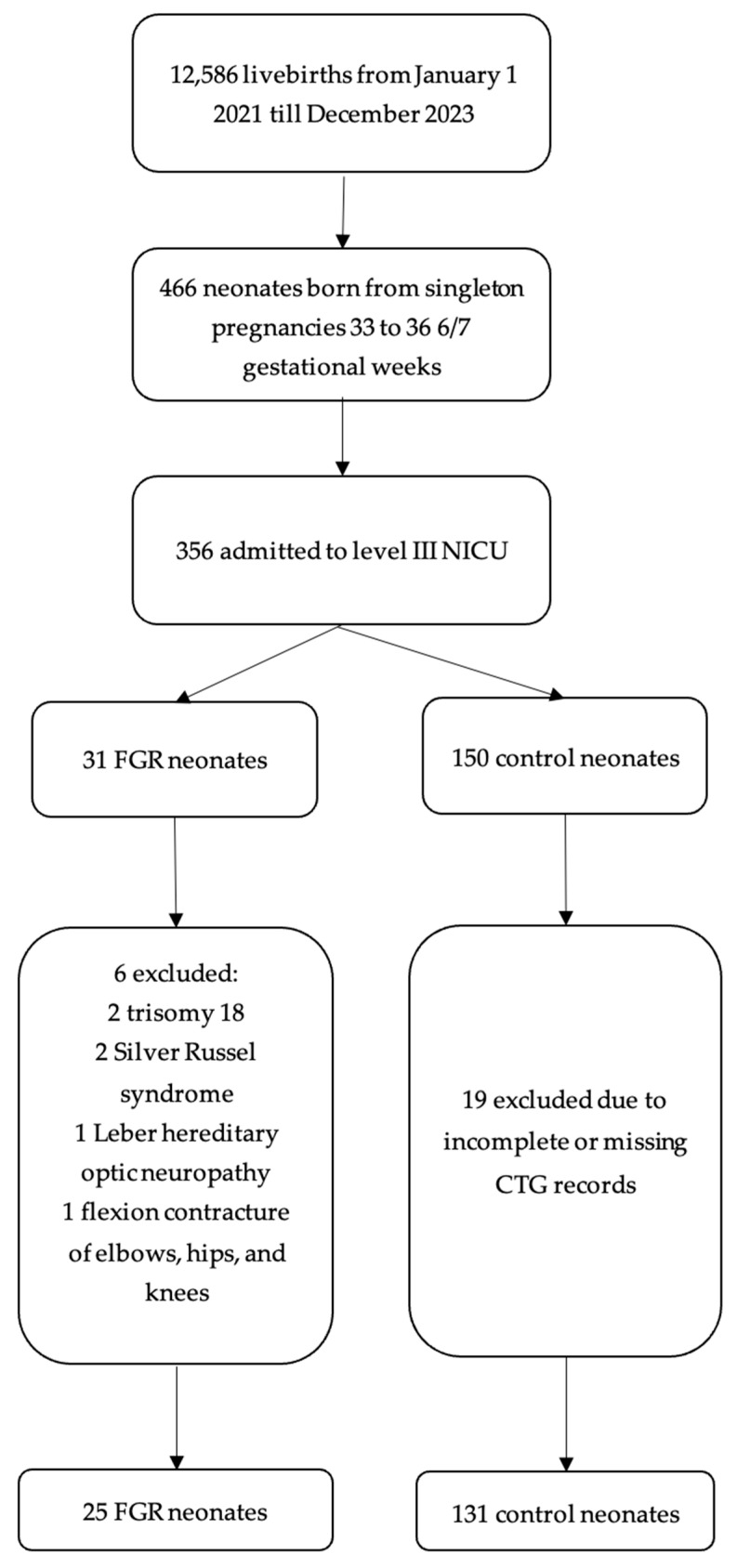
Flow diagram of studies selection.

Inclusion criteria for study cases consisted of all moderate to late preterm neonates from singleton pregnancies with diagnosed FGR born in three years in a single-center study. Control cases were neonates with the same gestational week as the study case, with a birth weight > 10th percentile, who were admitted to the NICU. FGR was defined by size, as follows: abdominal circumference (AC) or estimated fetal weight (EFW) < 10th percentile [26,27]. Exclusion criteria for study and control cases included chromosomal abnormalities, metabolic diseases, hereditary diseases, and transfers to another medical center. According to the inclusion criteria, a total of 31 FGR cases were identified, six were excluded, leaving 25 FGR cases in the case study group, highlighting that no FGR case was found in the 32nd gestational week (Figure 1). Sample size was calculated using the NICU population of 356 neonates, ensuring statistical significance with a significance level of 0.05, an 85% confidence interval, and a 5% standard error. As we could not single out data by gestational week, we selected control cases through simple random sampling using a protocol number from the paper data list of all neonates admitted to the NICU. If randomization selected a case with an unmatched gestational week, the subsequent case with a matched gestational week would be chosen. A total of 131 neonates were enrolled as the control group.

### 2.2. Fetal Heart Rate Monitoring

FHR monitoring was performed by a Philips Avalon-FM30 fetal monitor device. One gynecologist specialist who was blind to neonatal outcomes analyzed CTG traces. Intrapartum cardiotocography was analyzed according to the three-tiered classification system of fetal heart rate patterns of the American College of Obstetricians and Gynecologists, the Society for Maternal-Fetal Medicine, and the United States National Institute for Child Health and Human Development, and results were divided into the following three groups: Category I, II, and III [28].

### 2.3. Neonatal Outcome

Need for assistance after birth was defined as an intervention within the first 15 min of life, such as positive pressure ventilation, intubation, or chest compressions. RDS was defined using ICD 10 code P22.0 in discharge records, based on respiratory distress symptoms appearing within the first 24 h of life and the need for FiO2, following European guidelines for the management of the RDS [29]. Noninvasive respiratory support (NRS) modalities include the usage of nasal continuous positive airway pressure (CPAP), bi-level CPAP (BiPAP), high flow nasal cannula (HFNC), nasal intermittent positive pressure ventilation (NIPPV), and synchronized nasal intermittent positive pressure ventilation (SNIPPV). Vasoactive medications include dopamine, dobutamine, and noradrenaline and were used in the treatment of hypotension if the neonate had shown signs of poor perfusion. We monitored blood pressure noninvasively using a blood pressure cuff to measure systolic, diastolic, and mean arterial pressure (MAP) (typically hourly). Hypotension was defined as an MAP less than the gestational age of the neonate on the first day of life. In our NICU, the first-line therapy for neonatal hypotension is volume expansion through a 10 mL/kg intravenous bolus of 0.9% saline, repeated up to two times. In neonates who did not show improvement in blood pressure with volume expansion, vasoactive medications were employed. Our NICU prefers the use of dopamine, followed by noradrenaline or dobutamine. In our study, neonates were given only dopamine; no neonates received noradrenaline or dobutamine. Fresh frozen plasma transfusions in our NICU are administered to neonates with abnormal coagulation tests (mostly prothrombin time < 20%), active hemorrhage, or hypovolemia. The decision to administer FFP was made by the clinician. In our study, there were no cases of active hemorrhage; therefore, abnormal coagulation tests and hypovolemia were the indications for the administration of FFP. The surveillance and timing strategies of FGR neonates in our study were performed according to the ACOG Practice Bulletin: Antepartum Fetal Surveillance, Fetal growth restriction, except the diagnosis for oligohydramnios by amniotic fluid index (AFI) < 5 cm [30,31]. For pregnant women between 24 0/7 weeks and 33 6/7 weeks of gestation who are at risk of preterm delivery within 7 days, including those with ruptured membranes, a single course of corticosteroids was administered. The administration of corticosteroids in preterm pregnancies >34 0/7 weeks was the clinician’s decision. Indication for a repeated course of antenatal corticosteroids was found in pregnant women <34 0/7 weeks who were at risk for preterm delivery within 7 days and whose prior course of antenatal corticosteroids was administered more than 14 days previously.

### 2.4. Maternal Characteristics

During the years 2021, 2022, and 2023, there were 12,586 live births in the University Hospital Centre Split. Among them, there were 464 neonates born from singleton pregnancies from 33 to 36 6/7 gestational weeks, and 356 (76.7%) were admitted to the NICU. Our study included 156 neonates admitted to the NICU, of which 25 had FGR. There was no significant difference in gestational age, maternal age, gravity and parity, antenatal corticosteroids, tocolysis, antenatal antibiotics, breech, meconium stained fluid, PPROM, placenta previa, placental abruption, GBS+, liver disorders in pregnancy, diabetes mellitus in pregnancy, thrombophilia, and hypothyroidism during pregnancy between the two groups (*p* > 0.05) (Table 1). Significant differences existed between mode of birth, three-tiered FHR categorization, oligohydramnios, hypertensive disorders in pregnancy, and COVID-19 during pregnancy (*p* < 0.001). In the FGR group, the pregnant women were more likely to have hypertensive disorders, COVID-19, and oligohydramnios during pregnancy, increasing the category within the three-tiered FHR categorization and cesarean delivery (CD).

### 2.5. Statistical Analysis

The statistical analysis of data was performed using software SPSS version 26, producer IBM, USA. Categorical variables were reported as frequencies and percentages. Continuous variables were presented using the descriptive statistics method, expressed as mean and standard deviation (SD). Non-normally distributed data were reported as median and interquartile range (IQR). The Shapiro–Wilk test was applied to verify the normal distribution of the variables. Categorical variables were analyzed using Pearson’s chi-square test or Fisher’s exact test when the assumptions of the chi-square were not met. The students’ *t*-test was used to compare the means when the data were normally distributed. For non-normally distributed data, the Mann–Whitney test was employed. Correlations between the increasing category within three-tiered FHR categorization and maternal and neonatal outcome were evaluated using Sperman’s coefficient correlation. A linear regression model with the forward method was used to identify maternal variables and neonatal sex associated with the increasing category within three-tiered FHR categorization. Statistical significance was set at *p* < 0.05.

## 3. Results

### 3.1. Neonatal Outcome Characteristics

Dopamine was the only vasoactive medication used in our studied population due to hypotension. No neonate received dobutamine or noradrenaline. There were no significant differences in sex, first and fifth min APGAR score, need for assistance after birth, noninvasive respiratory support and duration, invasive ventilation and duration, surfactant administration, neonatal sepsis, dopamine use, and fresh frozen plasma transfusion (*p* > 0.05) (Table 2 and Table 3). There was a significant difference between birth weight, birth length, first min APGAR score, and LOS (*p* < 0.001). Neonates with FGR were likelier to have lower birth weight, lower birth length, lower first min APGAR score, and longer LOS. Only one neonate from the control group had hypoxic ischemic encephalopathy grade I, according to Sarnat staging criteria. Six neonates (3.84%) in our research received dopamine due to hypotension on the first day of life. Seven neonates (4.48%) received a fresh frozen plasma transfusion in the studied population due to abnormal coagulation tests and volume expansion on the first day of life. Endotracheal intubation was required in eight neonates, making an overall rate of endotracheal intubation of 5.1%. The other seven neonates needed only short-term ventilation using T-piece resuscitators in the delivery room. The overall rate of RDS was 7% in the whole study sample of neonates born from 33 to 36 6/7 gestational weeks.

### 3.2. Correlation Analysis Between Increasing Category Within Three-Tiered FHR Categorization and Neonatal Outcome in Moderate (n = 58) and Late (n = 98) Preterms

The study showed a positive correlation between sex with the increasing category within three-tiered FHR categorization (rho = 0.319, *p* = 0.015) in moderate neonates (Table 4). There were no correlations between gestational age with the increasing category within three-tiered FHR categorization in moderate or late neonates (rho = −0.021, *p* = 0.878; rho = −0.141, *p* = 0.165). First min APGAR score was negatively correlated with the increasing category within three-tiered FHR categorization in moderate and late neonates (rho = 0.343, *p* = 0.008; rho = −0.256, *p* = 0.Fifth min APGAR score was negatively correlated with the increasing category within three-tiered FHR categorization in late neonates (rho = −0.273, *p* = 0.007). The need for assistance within 15 min after birth was positively correlated with the increasing category within three-tiered FHR categorization in moderate neonates (rho = 0.311, *p* = 0.017).

The study found positive correlations between the increasing category within three-tiered FHR categorization and RDS in moderate neonates (rho = 0.298, *p* = 0.023), meaning that moderate neonates with the increasing category within three-tiered FHR categorization were more likely to have RDS (Table 5). Surfactant administration was positively correlated with the increasing category within three-tiered FHR categorization in moderate and late neonates (rho = 0.261, *p* = 0.048; rho = 0.260, *p* = 0.010). Noninvasive respiratory support and duration in days were positively correlated with the increasing category within three-tiered FHR categorization in late neonates (rho = 0.222, *p* = 0.028; rho = 0.218, *p* = 0.031). Invasive ventilation was positively correlated with the increasing category within three-tiered FHR categorization in moderate and late neonates (rho = 0.261, *p* = 0.048; rho = 0.260, *p* = 0.010). Invasive ventilation duration in days was positively correlated with the increasing category within three-tiered FHR categorization in late neonates (rho = 0.260, *p* = 0.010).

Dopamine use was positively correlated with cardiotocography, meaning that moderate and late neonates with the increasing category within three-tiered FHR categorization were more likely to need dopamine use (rho = 0.342, *p* = 0.008; rho = 0.212, *p* = 0.036) (Table 6). The need for fresh frozen plasma transfusion was positively correlated with cardiotocography, meaning that moderate and late neonates with the increasing category within three-tiered FHR categorization were more likely to need FFP transfusion (rho = 0.343, *p* = 0.008; rho = 0.212, *p* = 0.036).

Logistic regression has shown that the presence of oligohydramnios increases the probability for non-normal categories by 7.274 times within three-tiered FHR categorization (t = 3.040, *p* = 0.041) (Table 7). Male sex increases the probability for non-normal categories by 3.157 times within three-tiered FHR categorization (t = 9.009, *p* = 0.001).

## 4. Discussion

Our study reports a positive correlation between the increasing category within three-tiered FHR categorization and the need for assistance after birth in preterm neonates born from 33 to 36 6/7 gestational weeks in line with studies in term neonates [32,33]. Endotracheal intubation was required in eight neonates within the first 15 min, making an overall rate of 5.1% in the whole study sample. The incidence and timing of endotracheal intubation is poorly documented. In consistency with our data, Moya et al. published an overall rate of endotracheal intubation within the first 15 min of 5% in neonates born at 33–34 gestational weeks in 27 level III NICUs in the US, Canada, and Poland [34].

The overall rate of antenatal corticosteroid use in FGR pregnancies was higher than in control pregnancies (24% vs. 16.79%), but the difference was not statistically significant. Familiari et al. recently reported no beneficial effect of steroids on the short-term outcomes of fetuses with late FGR, nor any benefit for fetal lung maturation in preterm FGR pregnancies >32 weeks. More studies are needed to clarify the impact of antenatal corticosteroid administration in preterm FGR pregnancies after 32 weeks of gestation [35].

This study showed a positive correlation between the increasing category within three-tiered FHR categorization and RDS in moderate neonates. Abnormal FHR patterns are indicative of fetal hypoxia that may impact lung maturation and increase the risk of RDS, especially in premature neonates. Our study contributed to the existing literature by providing specific evidence using the three-tiered FHR categorization in neonates born from 33 to 36 6/7 gestational weeks. Fetal infection and/or inflammation are the most frequent pathophysiological processes that cause category II and even category III FHR patterns before development or in the absence of acidemia [36]. Due to the reduced secretion of surfactant in hypoxic and/or infectious/inflamed environments, surfactant administration, NRS, and IV correlated with the increasing category within three-tiered FHR categorization in our research. Previous results in term neonates report that category II was associated with an increased risk of respiratory morbidity [11,12,37]. Gromman reported a higher incidence of RDS in late preterm and term neonates born from emergency CD (13.3%) versus elective CS (8.98%) versus spontaneous vaginal delivery (2.92%), where fetal bradycardia (category III) was one of the causes for emergency CD [14]. Our study data emphasized no statistical difference in RDS between FGR and the control group, supporting the thesis that FGR accelerates lung maturation and surfactant production.

Six neonates (3.84%) in our research received dopamine due to hypotension on the first day of life. This is similar to the incidence reported in the Norwegian population database study that 2.7% of all NICU patients received inotropes, and 4.1% were <36 gestational weeks, respectively [38]. The United States data suggest a prevalence of dopamine and dobutamine use of 4.8% in preterm or LBW neonates [39]. To our knowledge, no data have been published regarding the incidence of dopamine use in Croatian NICUs. Hemodynamic disturbances in sick neonates are common, highly diverse in underlying pathophysiology, and dynamic. MAP is the most common parameter used to define hemodynamic instability clinically [40]. This study described a novel finding of a correlation between the increasing category within three-tiered FHR categorization and receiving dopamine. FHR not only reflects the behavior of the cardiovascular system but can also provide indirect information about the status of the autonomic nervous system, which plays an important role in maintaining adequate organ perfusion and in the regulation of blood pressure [41,42,43,44]. Golder has shown that neonates who develop clinically significant hypotension requiring treatment have impaired cardiovascular control that manifests within the first days after birth [22]. Therefore, changes in FHR could suggest a risk for the development of hypotension in preterm neonates born from 33 to 36 6/7 gestational weeks. Cohen reported that preterm FGR neonates display compromised heart rate variability on postnatal day 1, which may suggest increased vulnerability to circulatory instability and predispose them to systemic and cerebral hypoperfusion [45]. That is in contrast with our data, where there were no statistically significant differences in dopamine use in FGR and control cases. The gestational age difference could explain it due to the possible impact of prematurity on heart rate variability. The FGR group in the Cohen study ranged from 24 6/7 to 35 6/7, and our FGR group ranged from 33 to 36 6/7 gestational weeks [46,47,48]. Further studies with larger sample sizes that analyze FHR patterns and neonatal hypotension are necessary.

Seven neonates (4.48%) received fresh frozen plasma transfusion in the studied population due to abnormal coagulation tests and volume expansion on the first day of life. In a systematic review, Sokou demonstrated that the prevalence of FFP transfusions differs widely across centers globally, with the highest FFP transfusion prevalence of 12% and the lowest of 0.17%. The literature search indicated a wide variety of FFP practices around the world and the common pattern of prophylactic FFP administration in neonates with abnormal coagulation tests without evidence of active hemorrhage in most NICUs globally. The available evidence guiding FFP in neonates is fewer than the evidence guiding other blood product transfusions [21]. Our results demonstrated a correlation between the increasing category within three-tiered FHR categorization and FFP transfusions, which could be partially explained by coagulation dysfunction as a result of perinatal hypoxia. Diminished blood flow or oxygen to the fetus/neonate during the perinatal period can cause bone marrow and liver function impairment, leading to the impaired synthesis of clotting and fibrinolytic factors [49]. Our study highlighted no statistical differences between FGR and the control group. This is in contrast with Karapati’s systematic review, where FGR neonates presented with prolonged coagulation tests [50]. Larger prospective cohort studies are needed to elucidate differences in these results.

Oligohydramnios increases the probability for non-normal categories within three-tiered FHR categorization in our study population. Data about the association between oligohydramnios and EFM patterns in non-FGR pregnancies are conflicting [51,52,53]. Hasegawa et al. reported more early deceleration and prolonged deceleration during intrapartum monitoring in cases with oligohydramnios after 36 weeks of gestation, including FGR cases [54]. It is important to highlight that almost all oligohydramnios cases in our study were in the FGR group (6 of 7 cases). The oligohydramnios rate in our FGR group was 24%, similar to the rate in a large RCT study by Boers et al. in FGR neonates > 36 gestational weeks, yet higher than the rate of 7.94% reported by Yamoto in a retrospective review of 214 FGR cases in late preterm neonates [55,56]. Oligohydramnios present a risk factor for adverse outcomes in FGR pregnancies, and etiology may be due to placental underperfusion and, consequently, can be considered a risk factor for fetal hypoxia [31,57,58].

Evolving evidence suggests sex differences in postnatal complications among term and preterm neonates [59]. Data about associations between sex and EFM are contradictory. Our study demonstrated that male sex increases the probability for non-normal categories within three-tiered FHR categorization. Yohai et al. reported similar results in research of 682 singleton pregnancies that confirmed an independent association between male fetal sex and abnormal fetal heart monitoring during all stages of labor [60]. Bhide and Acharya reported that male fetuses show a significantly lower baseline FHR and greater variability as compared with female fetuses, highlighting that most neonates from the study were born at term. However, the absolute differences are small and may not be clinically significant [61]. In a study of 3639 term deliveries, male fetuses were at an increased risk for prolonged deceleration and repetitive variable deceleration [62]. One explanation of these differences is that the protocadherin gene expression in the brain affects brain anatomy and the chemistry of neuronal transmission. This expression is thought to be influenced by genes on the Y chromosome. Additionally, preterm females were found to have higher levels of catecholamines in blood than preterm males after exposure to asphyxia; this mechanism can explain the higher heart rates and better outcome of female infants compared to male infants [63,64,65]. Another possible explanation can be a higher rate of cord abnormalities in male neonates that had not been analyzed in our study [54].

### Strengths and Limitations of the Study

The primary limitation of our study is its retrospective nature. The small sample size, especially in the FGR group, consisting of only 25 neonates, is an obvious limitation of this study. Therefore, a large, multicenter, prospective cohort study is needed to confirm our results. Due to the complex and multifactorial etiology of hypotension and coagulation disorders in preterm neonates, it is challenging to elucidate the exact pathophysiological mechanism that completely explains the association between the increasing category within three-tiered FHR categorization and hypotension and coagulation disorders in preterm neonates. We tried to avoid interobserver variability in CTG interpretation by one observer’s CTG traces analysis, yet we need to emphasize the subjectivity of CTG interpretation as a possible limitation of the study. One potential limitation of this study is the exclusion of neonates with incomplete or missing CTG data, which may have introduced selection bias. These cases might systematically differ from those included in the analysis, potentially affecting the generalizability of our results. At the time of data collection, there were no formal national or institutional guidelines. Clinical decisions were therefore based on clinician judgment, commonly informed by international guidelines such as those from ACOG or NICE, depending on personal preference and training background. We acknowledge this as a limitation regarding standardization and external comparability. FHR category could not influence postnatal care because treatment decisions in our NICU are not under the influence of FHR category data. It is important to highlight the absence of the neurodevelopmental or long-term outcome in relation to three-tiered FHR categorization, highlighting only the short-term neonatal outcome of the study.

Due to reducing variability in clinical management and minimizing confounding factors, we found a single-center study to be a strength and advantage. Our study is one of few regarding the three-tiered FHR classification system and respiratory morbidity in moderate and late preterm neonates, including FGR neonates. It is also one of few studies that have analyzed the correlation of three-tiered FHR categorization and oligohydramnios in preterm neonates, including FGR neonates. To our knowledge, this is the first study that examined the correlation between three-tiered FHR categorization with dopamine use and fresh frozen plasma transfusion in neonates hospitalized in the level III NICU.

## 5. Conclusions

An increasing category within three-tiered FHR categorization may alert neonatologists to be highly suspicious of RDS, respiratory support, hypotension, dopamine use, and fresh frozen plasma transfusion in neonates born from 33 to 36 6/7 gestational weeks. Oligohydramnios and male sex increase the probability for non-normal categories within three-tiered FHR categorization. Despite its limitation, standard CTG is widely used in delivery rooms worldwide and may that suggest neonatologists vigilantly approach neonates with the worsening category within three-tiered FHR categorization.

## Figures and Tables

**Table 1:** Maternal characteristics.

	FGR (n = 25)	Control (n = 131)	*p* Value
Maternal age	32.20 (SD 5.859)	31.63 (SD 5.322)	*p* = 0.549 ^a^
Gestational age	35.00 (IQR 34.50–36.00)	35.00 (IQR 34.00–36.00)	*p* = 0.220 ^b^
Gravidy	1.00 (IQR 1.00–3.00)	2.00 (IQR 1.00–2.00)	*p* = 0.658 ^b^
Parity	1.00 (IQR 1.00–2.50)	2.00 (IQR 1.00–2.00)	*p* = 0.446 ^b^
Mode of birth/cesarean delivery	20 (80%)	52 (39.7%)	*p* < 0.001 ^c^
Antenatal corticosteroids	6 (24.0%)	22 (16.8%)	*p* = 0.390 ^c^
Antenatal antibiotics	12 (48.0%)	77 (58.8%)	*p* = 0.318 ^c^
Tocolysis	2 (8.0%)	16 (12.2%)	*p* = 0.420 ^d^
Breech	1 (4.0%)	8 (6.1%)	*p* = 0.561 ^d^
Hypertensive disorders in pregnancy	7 (28.0%)	7 (5.3%)	*p* < 0.001 ^c^
Diabetes mellitus in pregnancy	2 (8.0%)	7 (5.3%)	*p* = 0.439 ^d^
Liver disorders in pregnancy	0 (0.0%)	4 (3.1%)	*p* = 0.494 ^d^
Oligohydramnios	6 (24.0%)	1 (0.8%)	*p* < 0.001 ^d^
PPROM > 16 h *	1 (4.0%)	21 (16.0%)	*p* = 0.094 ^d^
Meconium-stained fluid	2 (8.0%)	4 (3.1%)	*p* = 0.246 ^d^
Placenta previa	1 (4.0%)	6 (4.6%)	*p* = 0.688 ^d^
Placental abruption	1 (4.0%)	8 (6.1%)	*p* = 0.561 ^d^
GBS+ **	1 (4.0%)	3 (2.3%)	*p* = 0.506 ^d^
COVID-19 during pregnancy	6 (24.0%)	10 (7.6%)	*p* = 0.013 ^c^
Thrombophilia	1 (4.0%)	7 (5.3%)	*p* = 0.296 ^d^
Hypothyroidism during pregnancy	2 (8.00%)	9 (6.9%)	*p* = 0.554 ^d^
Category I	2 (8.0%)	85 (64.9%)	*p* < 0.001 ^c^
Category II	6 (24.0%)	26 (19.9%)	
Category III	17 (68.0%)	20 (15.3%)	

n number of participants, ^a^ student’s *t*-test, ^b^ Mann-Whitney U test, ^c^ chi-square, ^d^ Fisher’s exact test, * preterm premature rupture of membrane, ** group B streptococcus positive vaginal and rectal swab test.

**Table 2:** Neonatal outcome characteristics.

	FGR (n = 25)	Control (n = 131)	*p* Value
Sex/male	14 (56%)	74 (56.49%)	*p* = 0.964 ^a^
Birth weight (g)	1750 (IQR 1545–1965)	2630 (IQR 2360–3020)	*p* < 0.001 ^b^
Birth lenght (cm)	43.00 (IQR 41.00–44.00)	47.00 (IQR 45.00–48.00)	*p* < 0.001 ^b^
First min APGAR score	8.00 (IQR 8.00–9.00)	9.00 (IQR 8.00–9.00)	*p* = 0.047 ^b^
Fifth min APGAR score	9.00 (IQR 8.00–9.00)	9.00 (IQR 8.00–10.00)	*p* = 0.161 ^b^
LOS * (days)	23.00 (IQR 18.50–34.00)	14.00 (IQR 11.00–20.00)	*p* < 0.001 ^b^

n number of participants, ^a^ chi-square test, ^b^ Mann–Whitney U test, * length of hospital stay.

**Table 3:** Neonatal outcome characteristics.

	FGR (n = 25)	Control (n = 131)	*p* Value
Need for assistance after birth	3 (12.00%)	12 (9.16%)	*p* = 0.444 ^a^
NRS *	5 (20.00%)	26 (19.85%)	*p* = 0.586 ^a^
NRS (days)	0 (IQR 0–0)	0 (IQR 0–0)	*p* = 0.889 ^b^
Invasive ventilation	1 (4.00%)	14 (10.69%)	*p* = 0.267 ^a^
Invasive ventilation (days)	0 (IQR 0–0)	0 (IQR 0–0)	*p* = 0.279 ^b^
Surfactant administration	1 (4.00%)	14 (10.69%)	*p* = 0.267 ^a^
Neonatal sepsis	2 (8.00%)	4 (3.05%)	*p* = 0.246 ^a^
RDS **	2 (8.00%)	9 (6.87%)	*p* = 0.554 ^a^
Dopamine usage	0 (0.00%)	6 (4.58%)	*p* = 0.344 ^a^
FFP *** transfusion	2 (8.00%)	5 (3.82%)	*p* = 0.312 ^a^

n number of participants, ^a^ Fischer’s exact test, ^b^ Mann–Whitney U test, * noninvasive respiratory support, ** respiratory distress syndrome, *** fresh frozen plasma.

**Table 4:** Spearman’s rho correlation between the increasing category within three-tiered FHR categorization and neonatal baseline characteristics.

	33–34 (n = 58)		35–35 (n = 98)	
	rho	*p*	rho	*p*
Sex	0.319	0.015	0.167	0.100
Gestational age	−0.021	0.878	−0.141	0.165
First min APGAR	−0.343	0.008	−0.256	0.011
Fifth min APGAR	−0.203	0.127	−0.273	0.007
Need for assistance after birth	0.311	0.017	0.008	0.387
LOS *	0.145	0.278	0.310	0.002

* Length of hospital stay.

**Table 5:** Spearman’s rho correlation between the increasing category within three-tiered FHR categorization and respiratory neonatal outcome.

	33–34 (n = 58)		35–36 (n = 98)	
	rho	*p*	rho	*p*
Respiratory distress syndrome	0.298	0.023	0.184	0.069
Surfactant administration	0.261	0.048	0.260	0.010
Noninvasive respiratory support	0.155	0.246	0.222	0.028
Noninvasive respiratory support (days)	0.200	0.133	0.218	0.031
Invasive ventilation	0.261	0.048	0.260	0.010
Invasive ventilation (days)	0.248	0.060	0.260	0.010

**Table 6:** Spearman’s rho correlation between the increasing category within three-tiered FHR categorization and neonatal medication outcome.

	33–34 (n = 58)		35–36 (n = 98)	
	rho	*p*	rho	*p*
Dopamine use	0.342	0.008	0.212	0.036
Fresh frozen plasma transfusion	0.343	0.008	0.212	0.036

**Table 7:** Logistic regression of non-normal categories within three-tiered FHR categorization, maternal characteristics, and neonatal sex.

		Parameter	t	*p*	95% CI
Model	(Constant)	0.514	0.642	0.212	
	Oligohydramnios	7.274	3.040	0.041	0.78–67.69
	Sex	3.157	9.009	0.001	1.49–6.69
	Breech	0.655	0.275	0.300	0.13–3.18
	Antenatal corticosteroids	0.437	1.556	0.106	0.12–1.60
	Antenatal antibiotics	0.912	0.051	0.411	0.41–2.03
	Tocolysis	3.406	2.388	0.061	0.72–16.12
	Hypertensive disorder in pregnancy	2.650	1.955	0.081	0.08–6.67
	Diabetes mellitus in pregnancy	0.653	0.289	0.296	0.14–3.10
	Liver disorders in pregnancy	0.877	0.013	0.454	0.09–8.28
	PPROM * > 16 h	0.245	0.478	0.245	0.23–2.03
	Meconium-stained amniotic fluid	1.892	0.393	0.2655	0.26–13.88
	Placenta previa	0.749	0.067	0.398	0.08–6.67
	Placental abruption	2.142	0.877	0.175	0.43–10.55
	GBS+ **	1.039	0.001	0.486	0.11–9.45
	COVID-19 during pregnancy	2.147	1.567	0.105	0.65–7.10
	Thrombophilia	5.5 × 10^9^	0.000	0.500	0.00
	Hypothyroidism in pregnancy	0.569	0.545	0.230	0.13–2.54

Cox and Snell R^2^ 0.518, Nagelkerke R^2^ 0.212, correct classification 67.3%, * preterm premature rupture of membranes, ** group B streptococcus positive vaginal and rectal swab test.

## Data Availability

The data presented in this study are available on request from the corresponding author due to privacy.

## Abbreviations

The following abbreviations are used in this manuscript:

NICU	Neonatal intensive care unit
FHR	Fetal heart rate
CTG	Cardiotocography
FGR	Fetal growth restriction
ICD	International Classification of Diseases
RDS	Respiratory distress syndrome
NRS	Noninvasive respiratory support
IV	Invasive ventilation
LOS	Length of hospital stay
FFP	Fresh frozen plasma
HIE	Hypoxic ischemic encephalopathy
AC	Abdominal circumference
EFW	Estimated fetal weight
CPAP	Nasal continuous positive airway pressure
BiPAP	Bi-level nasal continuous positive airway pressure
HFNC	High-flow nasal cannula
NIPPV	Nasal intermittent positive pressure ventilation
SNIPPV	Synchronized nasal intermittent positive pressure ventilation
MAP	Mean arterial pressure
AFI	Amniotic Fluid Index
ACOG	American College of Obstetricians and Gynecologists
PPROM	Preterm premature rupture of membranes
GBS+	Group B streptococcus positive vaginal and rectal swab test
LBW	Low birth weight
NICE	National Institute for Health and Care Excellence
